# Combination of Magnetic Lignocellulosic Particles, High-Density Polyethylene, and Carbon Black for the Construction of Composites with Tunable Functionalities

**DOI:** 10.3390/polym10010009

**Published:** 2017-12-22

**Authors:** Jingfa Zhang, Haowei Wang, Haigang Wang, Dan Xing, Zhijun Zhang, Qingwen Wang

**Affiliations:** 1 Key Laboratory of Bio-Based Materials Science and Technology (Ministry of Education), Northeast Forestry University, Harbin 150040, China; zjf2010dl@126.com (J.Z.); WHW5842679135whw@163.com (H.W.); zzj_1003@163.com (Z.Z.); 2 Beiguo Intellectual Property Service Co., Ltd., Harbin 150040, China; xingdan1991@gmail.com; 3 College of Materials and Energy, South China Agricultural University, Guangzhou 510642, China

**Keywords:** biocomposites, magnetic nanoparticles, magnetic properties, antistatic properties, mechanical properties

## Abstract

Biocomposites with unique functionalities for tailored applications are promising products for a sustainable future. In this work, a process concept of forming functional composites by combining of high-density polyethylene, carbon black, and magnetic lignocellulosic particles (wood flour) was demonstrated. The impacts of process parameters on morphologies, crystalline phase, and magnetic intensity of wood flour were identified. Magnetic, antistatic and mechanical properties of biocomposites were also evaluated. Lignocellulosic particles were encapsulated with magnetic nanoparticles, and the resulting composites exhibited tunable magnetic and antistatic properties. A noticeable feature is that magnetic nanoparticles were uniformly distributed in the matrices as a result of anchorage to lignocellulosic particles. Magnetic lignocellulosic particles and polymer resin had good compatibility. The resulting composites provided another opportunity for shielding materials, which could reduce the radiation in the living environment. These findings could provide a tunable strategy of the tailored use of lignocellulose-based composites in functional applications.

## 1. Introduction

The space we inhabit is full of electromagnetic waves. Many people believe that it exerts harmful effects on the human health [1,2]. Hence, the importance of electromagnetic interference (EMI) shielding is obvious [3]. Shielding materials are commonly used to reduce the damage on electronics instruments from EMI [2]. And a perception of a negative effect is common. So, there will be a huge market for shielding materials which can reduce the effect of EMI via absorption and reflection mechanisms, particularly in the field of architectural decoration and packaging materials.

Magnetic nanoparticles have drawn significant interest because of their distinct electronic, optical and magnetic properties. However, macro products do not have these properties [4]. Exhibiting superparamagnetic and microwave absorption, Fe_3_O_4_ nanoparticles are widely used in catalysts, microwave absorption, medicine, magneto fluids, and composites among others [5,6,7]. It is a common method to blend polymers and nanoparticles to prepare functional composites that possess attractive electrical, optical, or superior mechanical properties [8,9]. However, there is a challenge in using higher content of magnetic nanoparticles in composite. The nanoparticles can aggregate and reduce the mechanical properties of the resulting composites [10].

In addition, Fe_3_O_4_ nanoparticles can be used to prepare magnetic wood. Magnetic wood, which was firstly proposed by Hisao Oka, exhibits magnetic properties despite the fact that it was essentially wood [11]. Magnetic wood had been produced via impregnation and coating techniques [12]. Recently, chemical precipitation has been employed in the preparation of a magnetic wood composite by in situ coprecipitation of magnetic particles in the wood surface. In addition, the morphology, crystal structures, and magnetic characteristics of modified wood samples were examined, as well as their magnetic characteristics [13,14,15,16,17].

It would be interesting to see if we can combine the magnetic wood flour and polymer to manufacture biocomposites with magnetic and antistatic properties. Biocomposites have been gaining increasingly widespread applications because of the desirable properties, including non-toxic, water-fast, and environment-friendly characteristics, as well as low cost [18,19]. Recently, a great number of biocomposites with functional properties such as, fire retardant and antistatic properties, were studied [20,21]. Although many studies have been conducted on antistatic biocomposites [22,23], tunable biocomposites with magnetic properties are rarely investigated. Magnetic properties are needed for biocomposites being used as an EMI shielding materials. Biocomposites with magnetic and conductive properties can be used in floors and wallboards in bedrooms, equipment rooms, or electronic workshops.

Antistatic agents and conductive particles are typically used for improving antistatic performance for biocomposites [22,24,25]. A previous study indicated that carbon black and expandable graphite can enhance the electrical conductivity of wood flour/polypropylene composites. A large amount of carbon black particles was used to form a conductive network and reduce the electrical resistivity of composites. However, adding a large amount of carbon black can negatively affect the mechanical performance of composites because of an aggregation [23].

It is well known that Fe_3_O_4_ can be used in magnetic materials, electric conductors, and shielding materials. If wood flour covered with Fe_3_O_4_ nanoparticles and polymer are combined, the Fe_3_O_4_ nanoparticles will also form a conductive network in the resulting composites. Meanwhile, the magnetic Fe_3_O_4_ nanoparticles will be evenly dispersed in the resulting composites. The resulting biocomposites can exhibit magnetic properties as well as antistatic properties, which are suitable as shielding materials.

In the current study, wood flour was modified with γFe_2_O_3_ iron oxide nanoparticles (Fe_3_O_4_/Fe_2_O_3_) via in situ coprecipitation reactions of Fe^2+^ and Fe^3+^ in the solution. Magnetic biocomposites (magnetic wood flour/HDPE composites) were then prepared by combining the modified wood flour and high-density polyethylene (HDPE) filled with carbon black. The morphology and crystalline phase of the wood flour were characterized. In addition, the morphology, magnetic quality, electrical resistivity, and mechanical properties of the resulting composites were subsequently evaluated. The synergistic effect of carbon black and iron oxide nanoparticles on the antistatic property of the resulting composites was also determined.

## 2. Materials and Methods

### 2.1. Materials

With the use of a hammer mill, wood flour (40–80 mesh) was prepared from poplar veneer grown in Heilongjiang, China. HDPE pellets (5000S) were purchased from Daqing Petrochemical Co., Daqing, China. Its density is 0.954 g·cm^−3^ with a melt flow index of 0.7 g·10 min^−1^ (according to ASTM D1238). Maleic anhydride grafted polyethylene with a graft ratio of 0.88 wt % and a melt flow index of 4.85 g·10 min^−1^ was supplied by Nanjing Juxing Polymer Materials Co., Ltd. (Nanjing, China) Ferric chloride hexahydrate, ferrous chloride tetrahydrate, and sodium hydroxide purchased from Tianjin Kermel Chemical Reagent Co., Ltd. (Tianjin, China) were used as modifying agents.

### 2.2. Treatment of Wood Flour

On the basis of previous studies [26], 200 g of wood flour was impregnated in 1 L of 1.2 mol·L^−1^ iron aqueous solution composited with Fe^3+^ and Fe^2+^. The mole proportion of Fe^3+^ and Fe^2+^ was 1.6. The pH of the solution was adjusted to 10 by the dropwise addition of 2% sodium hydroxide solution. Then the prepared wood flour was removed, washed three times with deionized water, and finally dried at 80 °C for 10 h in a vacuum oven. The experiment was repeated 41 times. The wood flour’s weight of 8 kg was due to the loss of some alkali-soluble compounds during the treatment. The modified wood flour was cooled and sealed in plastic bags for later use.

### 2.3. Formation of the Magnetic Biocomposites

Dried untreated and treated wood flour were mixed with HDPE filled with carbon black at a specific ratio, as shown in Table 1. Prior to mixing, the HDPE and carbon black were blended using a corotating twin screw extruder with a screw blade measuring 30 mm in diameter and L/D = 36 (Nanjing Rubber and Plastics Machinery Co., Ltd., Nanjing, China). Then the wood flour and HDPE/carbon were compounded using the same extruder. The temperature for extrusion ranged from 150 to 165 °C over seven zones along the extruder barrel. The extruded material was pelletized into lengths of less than 2 mm and then formed with a 45-mm single screw extruder (Nanjing Rubber and Plastics Machinery Co., Ltd., Nanjing, China). The temperature of the die was 165 °C, and that of the screw ranged from 150 to 170 °C.

### 2.4. Microstructure of Wood Flour and Biocomposites

The microstructures of the treated wood flour, untreated wood flour, and the resulting biocomposites were characterized using a scanning electron microscopy (SEM, JSM-7500F, Japanese Electronics Company, Tokyo, Japan). The resulting composites samples were frozen in liquid nitrogen and then shattered. The fractured surfaces were sputter-coated with platinum to render them conductive. The fractured surfaces were also examined using the same scanning electron microscope with an accelerated voltage of 10 kV.

### 2.5. X Ray Diffraction

The crystalline structure of the wood flour was characterized by X-ray diffraction (XRD, D/MAX 2200 Rigaku, Tokyo, Japan) operated with Cu *K*α radiation (γ = 1.5418 nm) at a scan rate (2θ) of 4°·min^−1^ ranging from 5° to 80°. The applied current was 30 mA with an accelerated voltage of 32 kV.

### 2.6. Fourier Transformed Infrared (FTIR)

FTIR spectroscopy was obtained using a Magna-IR 560 (Thermo Fisher Scientific, Nicolet, Waltham, MA, USA). For FTIR analysis, thin sample disks were prepared by grinding the magnetic wood flour and pressing them with potassium bromide.

### 2.7. Magnetic Properties

The magnetic properties of both wood flour and the biocomposites were evaluated using a vibrating sample magnetometer (Squid VSM, Quantum Design, San Diego, CA, USA). The magnetic hysteresis loops of the composites were characterized. The maximum intensity of the applied magnetic field was 10,000 Oe.

### 2.8. Antistatic Property

Electrical resistivity tests were conducted at an applied voltage of 1000 V. The volume resistance of each sample was measured five times using the GEST-121 resistance tester (Beijing Guance Jingdian Electric Equipment Co., Ltd., Beijing, China). The volume resistivity of each sample was calculated separately using the following formulas:

(1) $$\rho_{v}=R_{v}\frac{A}{h}$$

where ρ_v_ represents the volume resistivity (unit: Ω·cm), *R*_v_ is the volume resistance (unit: Ω), *A* denotes the effective area of the guard electrode (unit: cm^2^), and *h* is the average thickness of the samples (unit: cm). For the final ρ_v_, five samples are averaged.

### 2.9. Mechanical Tests

The tensile test was conducted in accordance with ASTM D638-2004 in the atmosphere environment. A crosshead speed of 5.0 mm·min^−1^ was set. Six parallel samples were tested for each processed product.

The samples with 80 mm × 13 mm × 4 mm size were subjected to the three-point blending test according the ASTM D790-2004. A crosshead speed of 2.0 mm·min^−1^ was used for the test. The tensile and flexural tests were operated using a universal mechanical testing machine (CMT5504, MTS Systems Co., Ltd., Eden Prairie, MN, USA). Six parallel samples were used in the flexural test.

The unnotched impact test was conducted in accordance with ISO179-2000. An impact instrument (CJ5 Chengde Testing Machine Co., Chengde, China) with an impact velocity of 2.9 m·s^−1^ was used. There were ten parallel samples in each set.

## 3. Results and Discussion

### 3.1. Characteristics of Magnetic Lignocellulosic Particles

**Figure 1:**
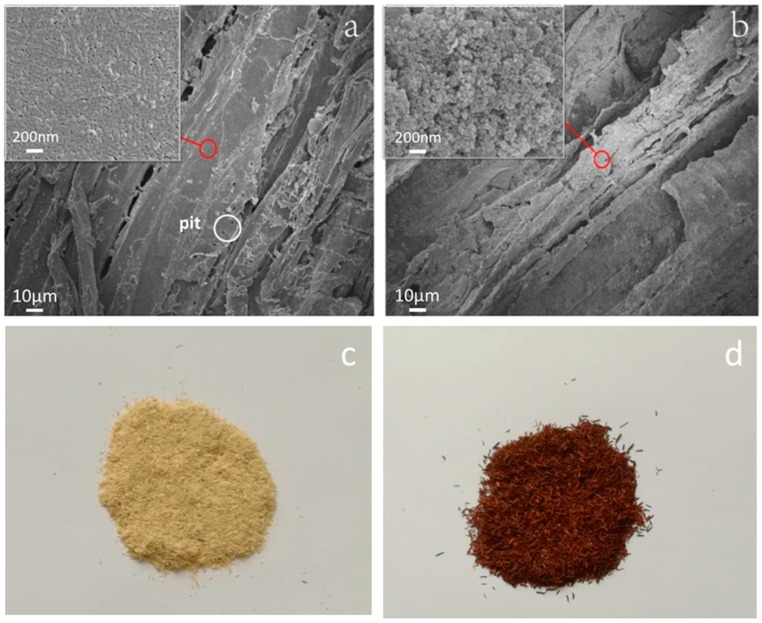
SEM images of untreated wood flour (**a**); magnetic wood flour (**b**); and the photos of untreated wood flour (**c**); magnetic wood flour (**d**).

The untreated wood flour had a smooth surface (Figure 1a), whereas the magnetic wood flour was covered with magnetic particles (Figure 1b). The pits on the surface of the untreated wood flour were clear (Figure 1a), meanwhile those of the treated wood flour were covered with magnetic nanoparticles. The high-magnification SEM image showed that the nanoparticles were densely stacked together. From Figure 1c,d, it can be seen that the color of wood flour had changed because that the wood flour was coated with magnetic nanoparticles. The result shew that nanoparticles evenly distributed on the surface of the wood flour. These nanoparticles were formed by chemical precipitation, potentially consisting of magnetic Fe_3_O_4_ and maghemite γ-Fe_2_O_3_ nanoparticles. The possible formation mechanism could be described as follows:

(2) $${\mathrm{Fe}}^{2+}\;+\;2{\mathrm{Fe}}^{3+}\;+\;8{\mathrm{OH}}^{-}\to {\;\mathrm{Fe}}_{3}O_{4}\;+{\;H}_{2}O$$

However, Fe_3_O_4_ was chemically unstable and sensitive to oxidation. Fe_3_O_4_ was transformed into maghemite (γ-Fe_2_O_3_) in the air because of the redox reaction [27].

**Figure 2:**
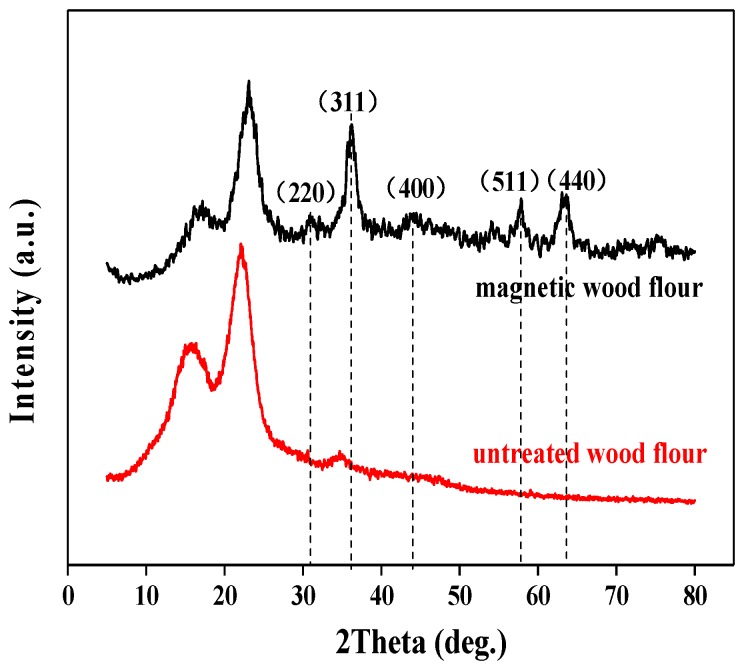
X-ray diffraction (XRD) patterns of the pure flour and the magnetic wood flour.

XRD diffraction patterns of pure flour and the magnetic wood flour are presented in Figure 2. Two characteristic peaks at 15° and 23° were observed for the pure wood flour, indicating the crystalline regions of cellulose in the wood flour [28,29,30]. After treatment, the diffraction peak position of cellulose hardly changed. This indicated that the crystalline form of the wood had not been changed in the process of the modification. Besides, for the treated wood flour, new diffraction peaks at five other angles were observed: 30°, 37°, 43°, 57°, and 62°. These peaks could be assigned to the diffractions of the (220), (311), (400), (511), and (440) planes of magnetite Fe_3_O_4_, respectively. However, owing to the similarity in the spinel structures of Fe_3_O_4_ and γ-Fe_2_O_3_, XRD could not effectively differentiate between the two phases.

**Figure 3:**
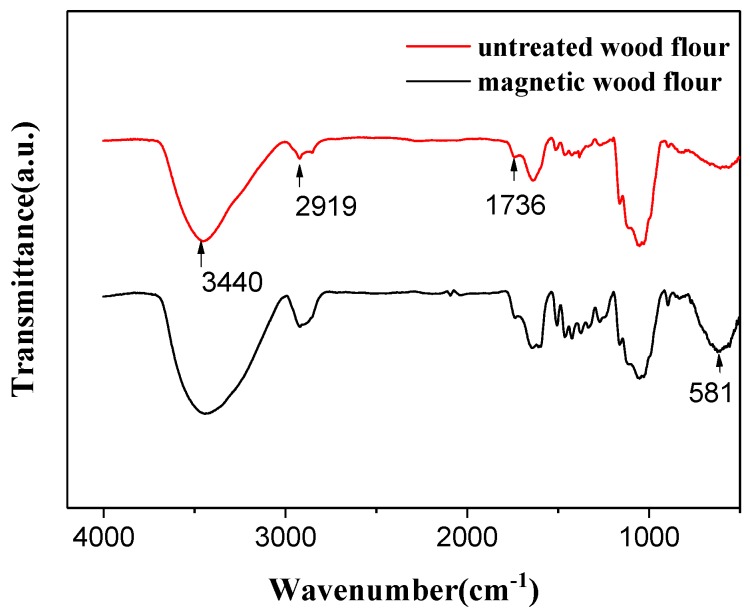
FTIR curves of the pure wood flour and the magnetic wood flour.

Figure 3 shows the FTIR spectra of the untreated wood flour and magnetic wood flour. 3440, 1736, 1637 and 1056 cm^−1^ were the characteristic absorption bands of wood. For the magnetic wood, the band of 1736 cm^−1^ shifted to 1730 cm^−1^, indicating a physicochemical interaction between wood flour and magnetic nanoparticles. The characteristic band at 581 cm^−1^ was arises from the stretching vibrations of Fe–O bonds [17,31].

**Figure 4:**
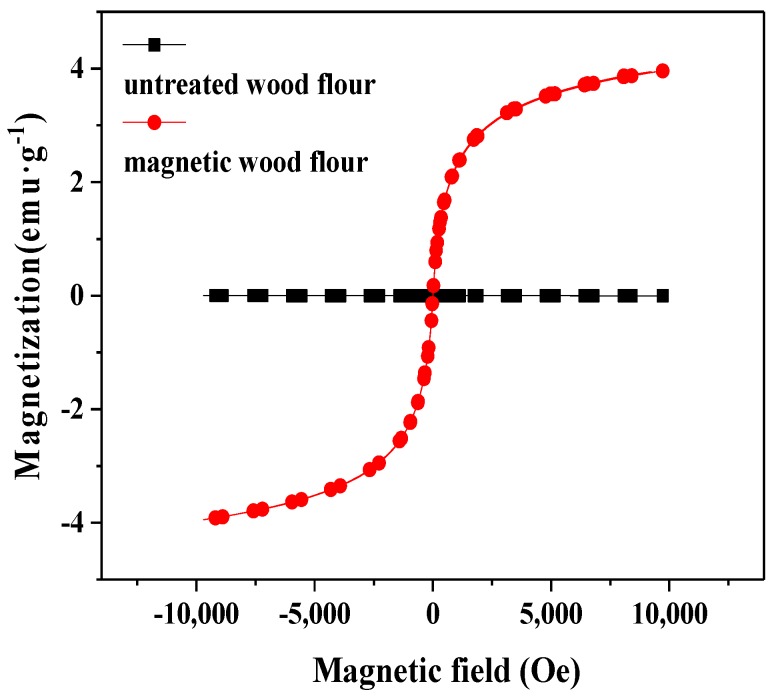
Magnetic hysteresis curves of the pure wood flour and the magnetic wood flour.

The magnetic property of the wood flour was presented in Figure 4. As shown, the hysteresis curve of the pure wood flour was a straight line near the zero point, which indicated the lack of magnetic contribution of the untreated wood flour. By contrast, the magnetic wood flour exhibited a typical ferromagnetic behavior. The magnetic intensity of the treated wood flour increased sharply with the increase in the magnetic field. As the magnetic field intensity continues to increase, the magnetic intensity of the treated wood flour increased gradually, reaching the saturation magnetization (*M_s_*) of 4 emu·g^−1^ at a magnetic field of 8000 Oe. Both coercivity and remanence of the magnetic wood flour were zero, indicating that the magnetic wood flour exhibits excellent superparamagnetism at room temperature. However, the *M_s_* value of the magnetic wood flour was considerably smaller than those of the corresponding pure Fe_3_O_4_ (63 emu·g^−1^) and γ-Fe_2_O_3_ (50 emu·g^−1^) nanoparticles synthesized by coprecipitation [32,33]. The reduction in *M_s_* could be attributed to the small number of magnetic nanoparticles precipitated on the wood flour surface.

### 3.2. The Structural and Surface Morphology of the Magnetic Biocomposites

**Figure 5:**
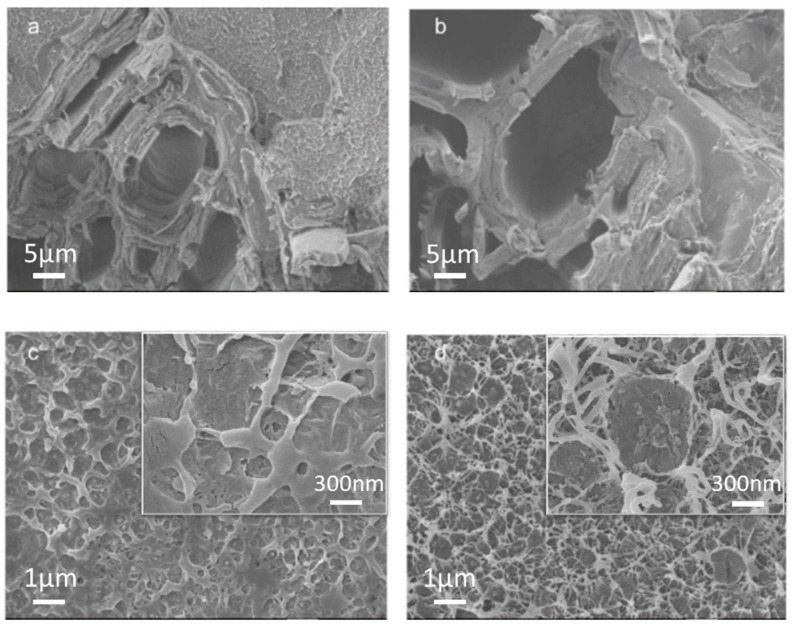
SEM images of original biocomposites (MWPC0) (**a**,**c**); magnetic biocomposites (MWPC20) at different magnifications (**b**,**d**).

**Figure 6:**
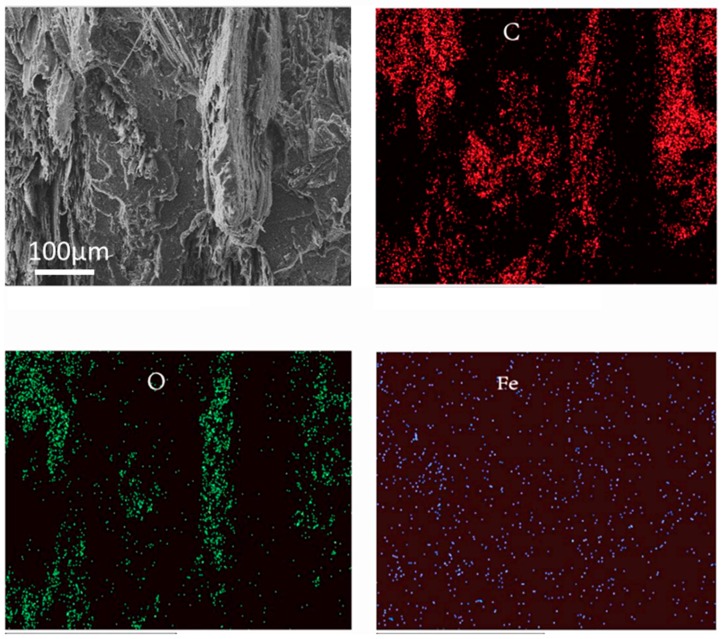
Energy dispersive spectroscopy (EDS) spectrum of the magnetic biocomposites (MWPC40).

Wood flour and matrix resin exhibited good compatibility because of the interfacial compatibilizer. The interface between magnetic wood flour and HDPE was similar to that between wood flour and HDPE (Figure 5a,b). However, there was no clear interface between magnetic lignocellulosic particles and HDPE. The improvement in interface compatibility was due to the surface effect of nanoparticles [34,35]. The fracture surface of the matrix in the original biocomposite was smoother than that in the magnetic biocomposites (Figure 5c,d). This difference was attributed to the rigidity of the magnetic nanoparticles, which changed the failure mode of the matrix resin [36]. High-magnification SEM images revealed that the magnetic nanoparticles were dispersed in the matrix as they detached from the wood flour because of the intense shear force in the extrusion processing of composites. The energy dispersive spectroscopy (EDS) spectrum presented in Figure 6 showed the distribution of the chemical elements of the magnetic biocomposites: carbon, oxygen, and iron are denoted in red, green, and blue, respectively. The magnetic nanoparticles were evenly dispersed in the composites via mixing in a twin-screw extruder, indicating that no aggregation of magnetic nanoparticles occurred using this method. If the magnetic nanoparticles were mixed directly with polymer in the composites, aggregation could occur. In this study, the problem concerning the uniform dispersion of nanoparticles was well-solved.

### 3.3. Magnetic Property of the Biocomposites

**Figure 7:**
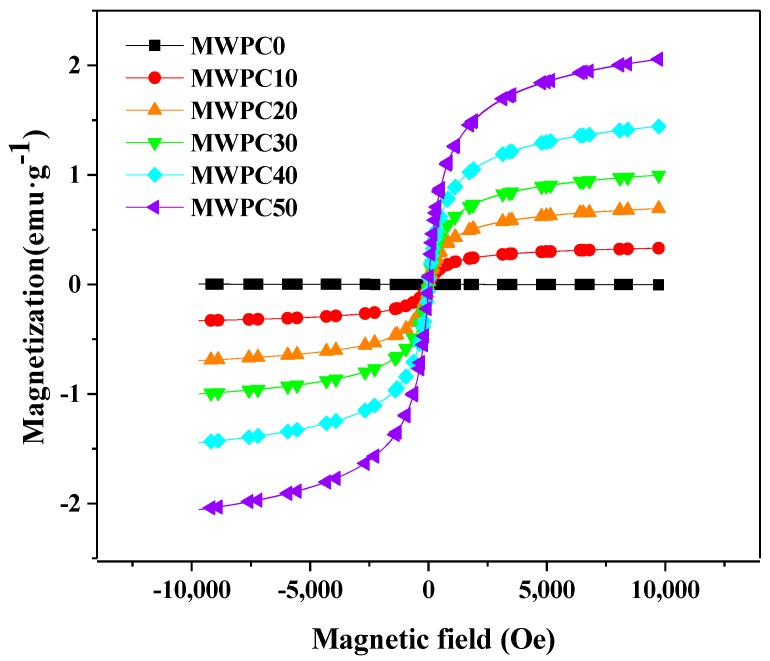
Magnetic hysteresis curves of the magnetic biocomposites.

The hysteresis curve of the magnetic biocomposites was similar to that of the magnetic lignocellulosic particle (Figure 7). The curve of MWPC0 was also a straight line near the zero point, indicating that the original biocomposites possess no magnetic property. The magnetic intensities of the magnetic biocomposites increased with an increase in the magnetic field. In addition, the hysteresis curve of the magnetic biocomposites shows no coercivity and remanence, suggesting that these composites also exhibit excellent superparamagnetic properties at room temperature. From the Figure 7, it can be seen that with the content of magnetic wood flour increasing, *M*_s_ of the magnetic biocomposites increased. This is because that the content magnetic nanoparticles in the resulting composites increased. *M*_s_ in the magnetic biocomposites was smaller than that in the magnetic wood flour because of the relatively less magnetic nanoparticles in the composites. The results shew that the magnetic intensity of the magnetic biocomposite could be regulated by changing the amount of magnetic lignocellulosic particles. Besides, the magnetic intensity of magnetic biocomposites varied with the applied magnetic field within a certain range.

**Figure 8:**
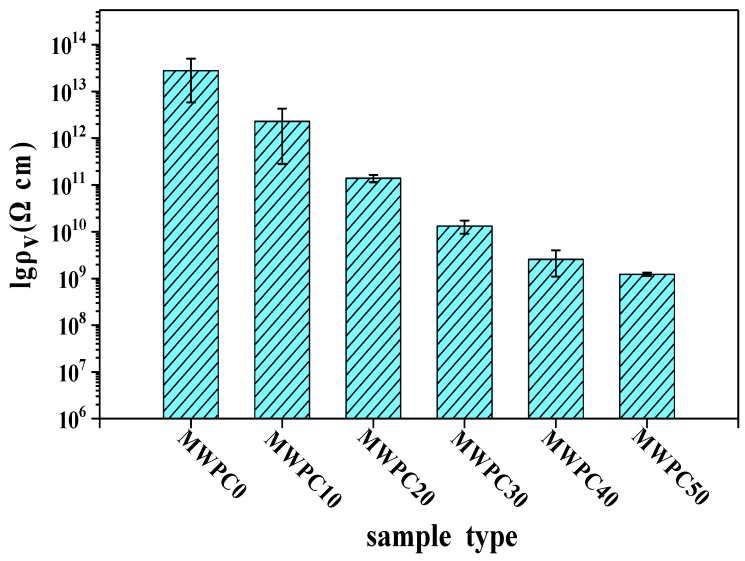
Volume resistivity of biocomposites.

**Figure 9:**
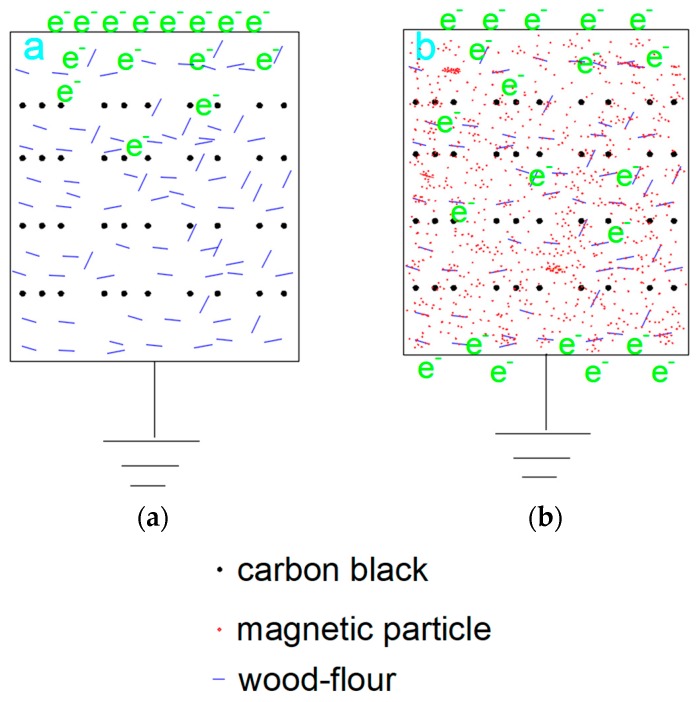
Assumed model of the synergistic effect of carbon black and magnetic lignocellulosic particles on electrical conductivity: (**a**) original biocomposites; (**b**) magnetic biocomposites.

The values for the volume resistivity of the composites were presented in Figure 8. The volume resistivity of WPC0 was about 10^13^ Ω·cm, which was consistent with that of WPC reported in the literature [23]. Carbon black was typically used as an antistatic agent for improving the antistatic performance of the composites. However, it caused only a slight reduction in the resistivity of the resulting composites samples because of a small content of carbon black. The mechanism was that such a small amount of carbon black could not create distances between the adjacent particles short enough to dissipate the electrostatic charges effectively [37]. In addition, a large amount of insulated wood flour prevented the electrons from transferring, as illustrated in the assumed model in Figure 9a. However, with the content of magnetic wood flour increasing, the electrical resistivity of the composite decreased due to the formation of conductive networks. Electrostatic charge was conducted between carbon black particles and magnetic nanoparticles. This model could be described as the assumed model in Figure 9b. The volume resistivity of MWPC50 was reduced to 10^9^ from 10^13^ Ω·cm for MWPC0 because of the synergistic effect of carbon black and the magnetic wood flour on the electrical conductivity of the resulting composites. The results revealed that the electrical resistivity of biocomposites can be tuned by varying the content of conducting particles.

### 3.4. Mechanical Properties of Magnetic Biocomposites

**Figure 10:**
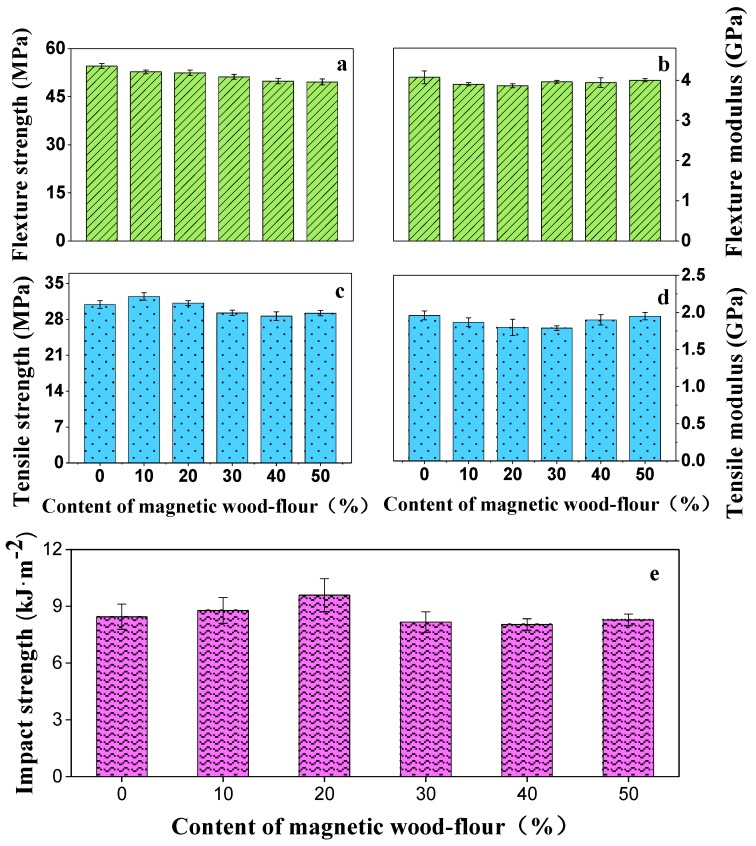
Mechanical properties of the magnetic biocomposites. (**a**) flexural strength; (**b**) flexural modulus; (**c**) tensile strength; (**d**) tensile modulus; (**e**) impact strength.

The magnetic wood flour exerted a slight or negligible effect on the flexural and tensile modulus of biocomposites. The flexural and tensile strength showed a tendency to decrease with an increase in magnetic wood flour content (Figure 10). The tensile strength of the resulting composite reduced to 29.22 from 30.89 MPa as well as the flexural strength reducing to 49.62 from 54.61 MPa. This decrease trend was influenced by many factors. Firstly, alkali added during the coprecipitation catalyzed wood flour degradation which resulted in a decrease in the strength of the magnetic wood flour [17,38]. The magnetic wood flour shortened due to the shear in extrusion. This could make the strength of the resulting composites decrease [39]. Secondly, the nanoparticles uniformly dispersed in the matrix could change the failure mode (Figure 5) and increase the strengths of biocomposites. There were other reasons, such as interface compatibility, the rheology properties of biocomposites and so on. Although the mechanical properties of composites are affected by such factors as alkali treatment, magnetic nanoparticle enhancement and interface improvement, the test results show that the mechanical properties are very insensitive to the magnetic wood flour.

With an increase in magnetic wood flour content in the composites, the impact strength of the magnetic biocomposites initially increased and then decreased. When the content of the magnetic wood flour reached 20%, the highest impact strength was obtained. This occurrence could be attributed to the mechanism underlying the rigid particle toughening of the polymer. The magnetic nanoparticles increased the impact strength of the matrix [40]. When the content of magnetic wood flour was more than 20%, a lot of low-strength magnetic wood flour may lead to a small decrease in impact strength of resulting composites. In conclusion, wood flour modification barely influenced the mechanical performances of the magnetic biocomposites when the content of the magnetic wood flour was less than 50%.

## 4. Conclusions

Magnetic biocomposites were prepared by the combined use of high-density polyethylene, carbon black and magnetic wood flour. Magnetic nanoparticles, which were anchored to wood flour, were uniformly distributed in biocomposites. The presence of nanoparticles resulted in improved interfacial compatibility. Magnetic nanoparticles and carbon black exhibited a synergistic effect on the electrical conductivity. A minor difference between the mechanical properties of magnetic biocomposites and original biocomposites was observed. The characteristics of biocomposites were tunable by adding varying amount of magnetic lignocellulosic particles. The biocomposites with tunable functionalities were shown to be an appropriate EMI shielding material, providing a possible strategy for reducing the impact of electromagnetic interference. They can be used as building decoration materials and packaging materials due to their advantageous properties.

## Figures and Tables

**Table 1:** Formulations of biocomposites for extrusion.

Sample Type	Untreated Wood Flour (wt %)	Magnetic Wood Flour (wt %)	HDPE (wt %)	MAPE (wt %)	Paraffin Wax (wt %)	Stearic Acid (wt %)	Black Carbon (wt %) *
MWPC0	50	-	42.68	4	1	1	1.32
MWPC10	40	10	42.68	4	1	1	1.32
MWPC20	30	20	42.68	4	1	1	1.32
MWPC30	20	30	42.68	4	1	1	1.32
MWPC40	10	40	42.68	4	1	1	1.32
MWPC50	-	50	42.68	4	1	1	1.32

* Note that the amount of carbon black was 3%, based on the dry weight of HDPE.
